# Current knowledge on the effects of environmental contaminants in early life nutrition

**DOI:** 10.3389/fnut.2023.1120293

**Published:** 2023-06-01

**Authors:** Maria E. Street, Anna-Mariia Shulhai, Roberta Rotondo, Giuliana Giannì, Carlo Caffarelli

**Affiliations:** ^1^Department of Medicine and Surgery, University of Parma, Parma, Italy; ^2^Unit of Pediatrics, University Hospital of Parma, Parma, Italy

**Keywords:** endocrine disrupting chemicals, breast milk, formula milk, early nutrition, gut microbiota, epithelial barrier, immunity, allergy

## Abstract

Breast milk represents the optimal source of feeding for newborns, in terms of nutritional compounds and as it provides immunological, metabolic, organic, and neurological well-being. As a complex biological fluid, it consists not only of nutritional compounds but also contains environmental contaminants. Formulas through production, contact with bottles and cups, and complementary feeding can also be contaminated. The current review focuses on endocrine-disrupting chemicals, and made-man xenoestrogens present in the environment and both commonly present in food sources, agricultural practices, packaging, consumer products, industry, and medical care. These contaminants are transferred by passive diffusion to breast milk and are delivered during breastfeeding. They mainly act by activating or antagonizing hormonal receptors. We summarize the effects on the immune system, gut microbiota, and metabolism. Exposure to endocrine-disrupting chemicals and indirect food additives may induce tissue inflammation and polarize lymphocytes, increase proinflammatory cytokines, promote allergic sensitization, and microbial dysbiosis, activate nuclear receptors and increase the incidence of allergic, autoimmune, and metabolic diseases. Breast milk is the most important optimal source in early life. This mini-review summarizes current knowledge on environmental contaminants and paves the way for strategies to prevent milk contamination and limit maternal and infant exposure during pregnancy and the first months of life.

## Introduction

The developing immune system can be dysregulated by environmental agents in early life. The goal of this review is to provide the current knowledge related with exposure to some environmental contaminants with nutrition from breast milk and formula milk to feeding with particular emphasis in infancy. We focus on the effects of endocrine-disrupting chemicals (EDCs) and indirect food additives.

### Endocrine-disrupting chemicals

Breastfeeding is the optimal natural process of feeding from the first hour after birth (1). Nutritive and non-nutritive breast milk compounds contribute to a child’s well-being, protect against infectious diseases, and promote immune and organ maturation, decrease the risk of developing obesity and type 2 diabetes, allergy, cardiovascular diseases, gastrointestinal, ear, and respiratory tract disorders, and on mental and behavioral health in both childhood and adulthood (1–5). Breast milk also contains a maternal microbiome community that colonizes the infant’s gut which is crucial for the infants’ health (2, 6). The presence of innate lymphoid cells in breast milk which share functions with T cells and play an important role in adaptive immunity and maturation of infants’ gut and microbiota, has been defined in recent years (7). Epigenetic regulation is performed by exosomal microRNA transport through breast milk also; these are then taken up by the intestinal epithelial cells (2, 3). Breast milk contains any contaminants to which the mothers are exposed (8). Among these, EDCs, made-man environmental chemicals, are present in food sources, consumer products, manufactured products, etc. EDCs are exogenous substances or mixtures that alter the function(s) of the endocrine system and consequently cause adverse health effects in an intact organism, or its progeny or (sub)populations (9). EDCs may bioaccumulate and bioamplificate in the body, and may be mobilized during the energetically-expensive periods of pregnancy or lactation (10). Prenatal EDCs can be transferred to the infant through transplacental absorption *in utero*, postnatally, through colostrum/breast milk (10, 11). Transfer of EDCs in breast milk occurs by passive diffusion through the membrane that separates the blood flowing in capillaries from the alveolar epithelial cells of the breast (2, 3). Studies have shown that breast milk is also an important matrix for biomonitoring exposure to contaminants in early life (3, 4, 10–13). Rovira et al. (14) found 31 organic contaminants and 14 toxic and essential elements in breast milk samples stored in a biobank in a Spanish cohort of nursing mothers. Interestingly, some compounds were higher in breast milk samples from low-income mothers as dichlorodiphenyltrichloroethane (DDT) and dichlorodiphenyldichloroethylene (DDE) (14). Differences were also seen in primiparous mothers compared with multiparous. Higher levels of bisphenol A (BPA) in low-income pregnant USA women were also found and were associated with adverse effects on offspring (15).

In infants, low or high concentrations of compounds cannot be adequately metabolized or excreted due to undeveloped physiology, anatomy, immature metabolizing enzymes and lower capacity to eliminate toxic compounds or because of the high sensitivity of target organs (9, 10, 16, 17).

Finally, EDCs concentrations in the human body, which depend on individual factors, environmental parameters related to diet, indoor and outdoor exposure, have an effect on the human microbiome (9, 11, 18, 19) also affecting immunity.

### Endocrine disrupting chemicals, their major effects and findings in breast and formula milk

EDCs are natural or synthetic substances that interfere with the synthesis, secretion, transport, metabolism, binding action, or elimination of natural hormones and are present in our daily life products and environment (9, 20, 21). Naturally occurring compounds with endocrine-disrupting potential are metals and metalloids, parabens, polyaromatic hydrocarbons (PAHs), and phytoestrogens. Man-made synthetic chemicals are commonly used in agricultural practices (pesticides, insecticides, and fungicides), packaging (food-storage materials and plastics), industry (solvents, flame retardants, preservatives, emulsifiers, and fracking chemicals), consumer products (household chemicals, cosmetics, flame retardants, building materials, children’s toys, electronics, and cookware), and medical care (birth control pills, biocides, intravenous bags and tubing, disposable gloves, and disinfectants) (9, 22). Some EDCs are xenoestrogens, others activate or antagonize hormonal receptors by direct binding or altering hormone receptor expression or signal transduction in hormone-sensitive cells, and most induce epigenetic changes (9, 10, 21, 23, 24). At the cellular level EDCs inhibit lysosome and mitochondria functions, causing DNA damage and UVB-induced damage through the production of reactive oxygen species and nitric oxide (25). Multigenerational effects of EDCs have been demonstrated in rodent studies up to four generations (26, 27). Endocrine disruptions involve all endocrine pathways, including effects on the placenta (Table 1).

Phthalate metabolites and BPA mimic endocrine nuclear receptors, modulate genes through epigenetic changes such as changes on DNA methylation, histone modifications, and effects on non-coding RNAs including micro RNA (miRNAs) expression and have been detected in breast milk in many studies (4, 15, 21, 25), and in infant formulas (16).

Maternal exposure to parabens has been associated with abnormal inflammatory cytokine levels in the blood in infants (24). Exposure to parabens leads to altered microbial composition, perturbed steroidogenesis, and induces oxidative stress and inflammation (19, 28). Parabens have been found in Chinese women’s breast milk and infant formula (28), and in Canadian women’s breast milk also (29).

Dioxins have estrogenic effects through the interaction of the dioxin- aryl hydrocarbon receptor (AhR) nuclear translocator complex with estrogen receptors (ER), which regulate in turn other nuclear receptors and there are few studies that have shown the presence of dioxin or dioxin metabolites in breast milk (13, 30).

Perfluoroalkyl substances (PFAS) have a cumulative toxic effect through the activation of nuclear receptors and by binding different protein receptors, and are limited studies about their presence in breast milk or formulas (9, 16, 17).

Pesticides can bind to ER and stimulate ER-dependent transcriptional activation and proliferation, inhibit androgen binding to the androgen receptor and its activation (31).

While EDCs have been detected in breast milk, studies have also shown that they can be present in infant formulas and contaminated food. Therefore, infants who are not breastfed may still be exposed to EDCs through their diet (6, 16–18, 28).

**Table 1:** Endocrine disrupting chemicals mechanism of action.

**Endocrine disrupting chemicals**						
**Mechanism of action**	**Nanoparticles**	**Bisphenol A**	**Phthalates**	**PFAS**	**Pesticides**	**Dioxins**
Mimic hormones	–	++	+	+	+	+
Stimulation receptor signaling	+	+	–	+	+	+
Inhibitition of receptor signaling	+	+	+	+	+	+
Stimulation or inhibition of hormone synthesis	+	+	+	+	+	+
Hormonal signal disruption	+	+	+	+	+	+
Changes in hormone receptor expression	+	+	+	+	+	+
Epigenetic changes	+	+	+	+	+	+

+, evidence of action; – stands for no known effect; ++, strong evidence; ±, there is some data on EDC effect. PFAS, Perfluoroalkyl substances.

## Gut microbiota

The gut microbiota (GM) represents the largest microbial community in the human body, estimated to be more than 1,014 bacteria associated with archaea, viruses, fungi, and protozoa (32, 33). GM is an “organ” in its own right and the human being should be considered as a “superorganism” consisting of the combination of *Homo sapiens* cells and microbial flora (34). The GM consists of anaerobic, facultative anaerobic and aerobic bacteria. Ninety percent is composed by Firmicutes and Bacteroidetes species (35).

The fetal human gut is physiologically sterile and is progressively colonized (36). At about 2 years of age the gut flora becomes similar to the adult one (37). The process colonization in newborns begins during delivery and is influenced by many factors including the mode of delivery (38, 39). As a vital and dynamic “microbial organ”, the GM plays a role in host well-being for digestive processes and nutrient absorption, growth and development, activation of the immune system to protect the host from pathogens (40–42). Dysbiosis correlates with health disorders, including metabolic alterations, neurodevelopmental disorders, inflammatory bowel disease, allergy, diabetes, obesity, cancer, infections and cardiovascular diseases (43, 44).

Breastfeeding modulates GM (45) through an entero-mammary pathway involving the transfer of different microbes from the mother’s gut to the baby through breast milk (46, 47) with a protective effect (48). It is well known that in contrast with formulas (42), breast milk contains complex human milk oligosaccharides that act as selective prebiotics in the colonization of the child’s gut, generating beneficial microbiota (49, 50). HMOs have shown to have a major impact on gut microbiota in breastfed infants, working as growth substrates for specific colonic bacteria, mainly belonging to the Bifidobacterium genu. These latter have a protective effect against inflammation and infection. Formula fed infants do not have HMOs, and their microbiota generally consists of other bacterial species, such as Enterobacteriaceae, Clostridia, and Staphylococci. This may have implications for future health, as a less diverse gut microbiota has been associated with an increased risk of health disorders (49, 50).

The breast-gut axis refers to the connection between breast milk and the development and maintenance of the gut microbiota in infants by compounds present in breast milk. It includes also a feedback loop between the gut microbiota and breast milk production. Studies have shown that the composition of breast milk changes over time, with variations in the levels of nutrients and bioactive components based on the infant’s needs. This suggests that the gut microbiota may communicate with the mammary gland, influencing breast milk composition in response to changes in the infant’s gut microbiota.

Breast-fed babies gut microbiota consists mainly of Bifidobacterium and Lactobacillus, and after breastfeeding has ended it becomes enriched with other species (Roseburia, Clotridium, and Anaerostipes) remaining the major driver in the development of adult microbiota. EDC increase the numbers of adult-like bacteria (49, 50).

### The effects of EDCs on the gut microbiota

The gastrointestinal tract is the main route of entry of EDCs. Their absorption in the gut is poor and they are transported by the peristaltic movement to the distal small intestine and cecum where the microbial flora metabolizes them directly, increasing or decreasing their toxicity. The portal circulation transports part of the EDCs to the liver where they are conjugated and excreted in the bile, thus entering the small intestine again where they undergo further deconjugation by the local microbiota, restoring the original compounds or producing new toxic metabolites (51).

Microbiota disrupting chemicals is the term used to group substances that alter these gut microbial pathways. EDCs can be metabolized by microbiota in a bidirectional interaction to biologically active or inactive forms, and EDCs can prompt the proliferation and growth of certain bacteria. These changes can lead to disturbances in different host systems. *In vitro* and *in vivo* models have shown that several EDCs promote dysbiosis or inhibit bacterial growth (51).

Dysbiosis and immune system dysfunction precede the development of the obese phenotype in mice perinatally exposed to BPA (52, 53). Structural changes in the GM exposed to BPA with diet were similar to those found in mice on high-fat and sucrose diets that were correlated with metabolic disorders and inflammatory bowel disease (54, 55). Exposure to polychlorinated biphenyls (PCBs) during growth can induce dysbiosis and epithelial permeability defects in the ileum and colon (56), specifically, an increased Bacteroidetes -to- Firmicutes ratio (57).

Three rodent studies showed that exposure to pesticides induced dysbiosis in the microbiota and inflammation (58–60). Few data are available regarding the effects of parabens on the microbiota (19). Exposure to triclosan has been shown to induce changes in the GM of rats (19, 61–64), and is associated with increased Bacteroidetes, and lipid accumulation (65, 66).

Phytoestrogens are also modulators of the GM and can in turn be metabolized (67). Their metabolites have stronger estrogenic activity than natural compounds and, due to microbiome variability, there are large differences in their effects among individuals (68, 69).

Dietary 2,3,7,8-tetrachlorodibenzofuran would alter the composition of the GM, shifting the ratio of Firmicutes to Bacteroidetes, triggering inflammation and modifying host metabolic homeostasis (70).

Moreover, the GM via surface molecules and metabolic products communicates with cells of the innate immune system. EDCs and dysbiosis can impair this communication and the function of the gut mucosal barrier (71–73) causing immune-related diseases (74). Among these EDCs the most important is BPA (75, 76).

Finally, EDCs in the diet and environment could influence other microbiota in different parts of the body other than the gut. Gonzalez et al. showed temporal changes in the milk microbiome of healthy Guatemalan mothers throughout the lactation period. A shift from Staphylococcus and Streptococcus species present at the beginning of lactation, to Sphingobium and Pseudomonas species found at the end of lactation was described. Interestingly, the species found in early lactation included commensal bacteria known to colonize the oral and intestinal tracts, whereas the species found in late lactation, showed common functional traits associated with the biodegradation of hazardous substances (77). Therefore, overall exposure to EDCs through foods can alter GM and activate pathways involved in the metabolism of EDCs favoring the development of different metabolic diseases. It remains unclear whether EDCs-induced metabolic disruptions in the host occur before changes in the microbiome or whether EDCs-induced changes in the microbiome cause metabolic disruptions (78).

### Indirect food additives, immune-mediated reactions and the epithelial barrier hypothesis

The “epithelial barrier hypothesis” (79) suggests that the epithelial barrier function can be disrupted by indirect food additives including nanoparticles, nano-microplastics, chemicals, enzymes and emulsifiers in processed food. Barrier impairment provokes dysbiosis (80) with the translocation of altered microbiota through the damaged barrier resulting in chronic tissue inflammation and polarization of lymphocytes toward specific phenotypes. These include chronic immune conditions like inflammatory bowel disease, systemic lupus erythematosus disease, and rheumatoid arthritis characterized by T helper(Th)1/Th17 or Th23 responses (81–84). Moreover, in predisposed individuals, barrier disruption in allergic diseases including atopic dermatitis (43), food allergy (85) and asthma (86, 87), predisposes to allergen penetration that differentiate Th2 cells leading to IgE production. Allergens can also trigger innate lymphoid cell 2 to activate a T2 response (88, 89). An impaired epithelial barrier can precede sensitization development (90, 91). On the other hand, a T2 inflammation can increase barrier damage.

#### Nanoparticles, metals and nano-microplastics

Nanoparticles <1,000 nm, both metals (titanium, silicon, and zinc) and lipids, impair the gastrointestinal barrier leading to changes in GM and inflammation (92). This may explain the increasing incidence of autoimmune diseases (84). *In vitro*, SiO2, TiO2 (93, 94), and ZnO nanoparticles translocate to the extracellular area. Nanoparticles can also bind to membrane macrophage receptors (95) inducing phagocytosis and/or activating the NLRP3 inflammasome pathway (92). Nano-microplastics (NMP) are ubiquitous and accumulate in tissues, including placenta (96). They can also carry harmful chemical pollutants. Although infant intake of microplastics released by infant bottles is high (97), studies on NMP safety are lacking in infants. It is hypothesized that microplastics may damage the epithelial barrier and modify the immune responses. Acrylate monomer microplastics for floor cleaning, irritated conjunctive and airways in adolescents (98). In mice, polystyrene microplastic ingestion provoked microbiota dysbiosis, decreased mucus secretion and damage of barrier function in the gut (99). In pregnant mice, polyethylene microplastics ingestion (100) changed GM, impaired barrier with inflammation. Polyethylene microparticles reduced dendritic cells and increased both IgA and helper/cytotoxic T cells ratio (101). So far, the impact on health is largely unknown.

#### Antiseptics & phthalates

Using pacifiers cleaned with chemical antiseptics but not with boiling water increased the risk of food allergy (95) suggesting that the hazard is not linked to altered oral microbiota. In infants whose pacifiers were cleaned by sucking, an altered composition of microbiota and a reduced frequency of allergic disorders were observed in comparison with other cleaning methods (102). Thus, it remains unclear whether oral antiseptic exposure may affect oral and gut microbiome. Moreover, antiseptics increase plasticizer release such as phthalates. Phthalate exposure can increase the frequency of asthma and allergic sensitization to aeroallergens (103). *In vitro*, phthalates increase production of proinflammatory IL-6 and IL-8 (104). However, vulnerability of children to phthalates should be confirmed since dietary exposure to phthalates in some studies was not a matter of concern (105).

#### Bisphenol A

BPA has been banned in baby bottles and children’s cups (106). However, it is used in teethers (107), in food and beverage containers to prevent metal corrosion and in polycarbonate plastics (108). *In vitro*, BPA disrupts the epithelial cell and induces Thymic Stromal Lymphopoietin production. In female mice, BPA exposure alters GM with decreasing Firmicutes and increasing pro-inflammatory Bacteroides species linked with b-cell autoimmunity resulting in type 1 diabetes development and exacerbation (109) and inflammatory bowel disease. In animal models, BPA impairs both the gut and airway barriers inducing chronic inflammation with innate immune system involvement. This may promote allergic sensitization and autoimmune diseases (76, 110, 111). Maternal bisphenol ingestion induced allergic lung inflammation in adults (112). These findings paved the way to studies on the role of BPA on allergic diseases in childhood. High urinary BPA levels in pregnancy (113), in preschool children (114), in school children (115) and in teenagers (116) were associated with preschool wheezing (113, 114), asthma (115, 116) and concomitant increase in IgE concentrations (114). However, urinary triclosan and propyl and butyl parabens but not BPA levels were associated with IgE to foods or inhalants in children (117). Bisphenol S does not safely replace BPA (118–120). Further studies are warranted to determine the effects of BPA on the immune system in infants.

#### Perfluoroalkyl substances

Perfluoroalkyl substances (PFAS) exposure occurs mainly through food products as they are contained in greaseproof paper and paperboard (121). They persist for years and accumulate in different tissues (122, 123), including breast milk (124). PFAS compounds have cumulative toxic effects including immunotoxicity (9, 125). Perfluorooctane sulfonic acid (PFOS), perfluorooctanoic acid (PFOA), perfluorononanoic acid (PFNA) and classes of long-chain perfluoroalkyl compounds have been banned because of safety concerns. However, short-chain PFASs are still marketed (126). Neonatal PFOA and PFOS levels were correlated with elevated IgA, IgM, IgG2, and lower IgE (127). Elevated estimated PFAS exposure during infancy induces lower diphtheria and tetanus antibody levels while the relationship is weak at 18 months and 5 years of age (128). Accordingly, infants with elevated PFAS levels in cord blood were at higher risk of respiratory tract infections from 1 to 5 years of age and had lower serum IgG concentrations (129). Prenatal exposure to PFOS and PFOA was also associated with higher prevalence of fever in young children (130). Conversely, blood PFOS and PFOA concentrations during pregnancy did not predispose to hospitalizations for infectious illness in childhood (131). Contrasting data have been provided regarding Th2 responses and asthma occurrence (132–134).

#### Pesticides

The dysregulation of the immune system caused by pesticides is unclear. In Inuit children, prenatal exposure to DDE and hexachlorobenzene increased otitis media frequency (135). However, prenatal, perinatal or postnatal exposure to DDE was not associated with respiratory infections (136, 137) or levels of lymphocytes and monocytes (137, 138) while it was inversely related with circulating eosinophils (138, 139). Conversely, in Ghana, a significant association between DDE and other pesticides and increased low respiratory infections in children aged 2 to 5 years was described (140).

#### Dioxins

Divergent data on the effect of PCBs have been provided in infants (141). Perinatal exposure to PCBs were not associated with respiratory infections at 12 months of age (136) and at 18 months (142). However, an increased risk of respiratory infection in the first 3 months of life prenatally exposed to PCB congeners was described (137). Lymphocytes and monocytes increased in prenatal exposure to CB-28, CB-52 and CB-101 congeners. Accordingly, PCB exposure in early childhood was associated with otitis media, chicken pox, bronchitis (143–145), and a lower prevalence of allergic reactions (143).

Higher maternal PCB exposure was associated with less wheeze and total polychlorinated dibenzodioxins (PCDDs), polychlorinated dibenzofurans (PCDFs) while PCB exposure was associated with coughing, chest congestion, and phlegm [108] at 42 months of age. Combined DDE and PCBs exposure was associated with otitis media (146).

Maternal hexachlorobenzene and PCBs but not DDE exposure was directly associated with asthma medication consumption in offspring (147) and no risk of allergic sensitization at 20 years of age (148). Perinatal dioxin exposure was inversely associated with the FEV1/FVC ratio at 7–12 years (149) and allergy, while there was an increase in Th cells and in T regulatory cells related to postnatal exposure (150). Maternal dioxin-like compounds were inversely related with cord blood IgE and wheezing in boys at 3.5 years of age and associated with wheezing in boys and girls at 7 years of age (151). PCB congeners increase serum AhR bioactivities (152) correlated with atopic dermatitis (153).

## Conclusion

**Figure 1:**
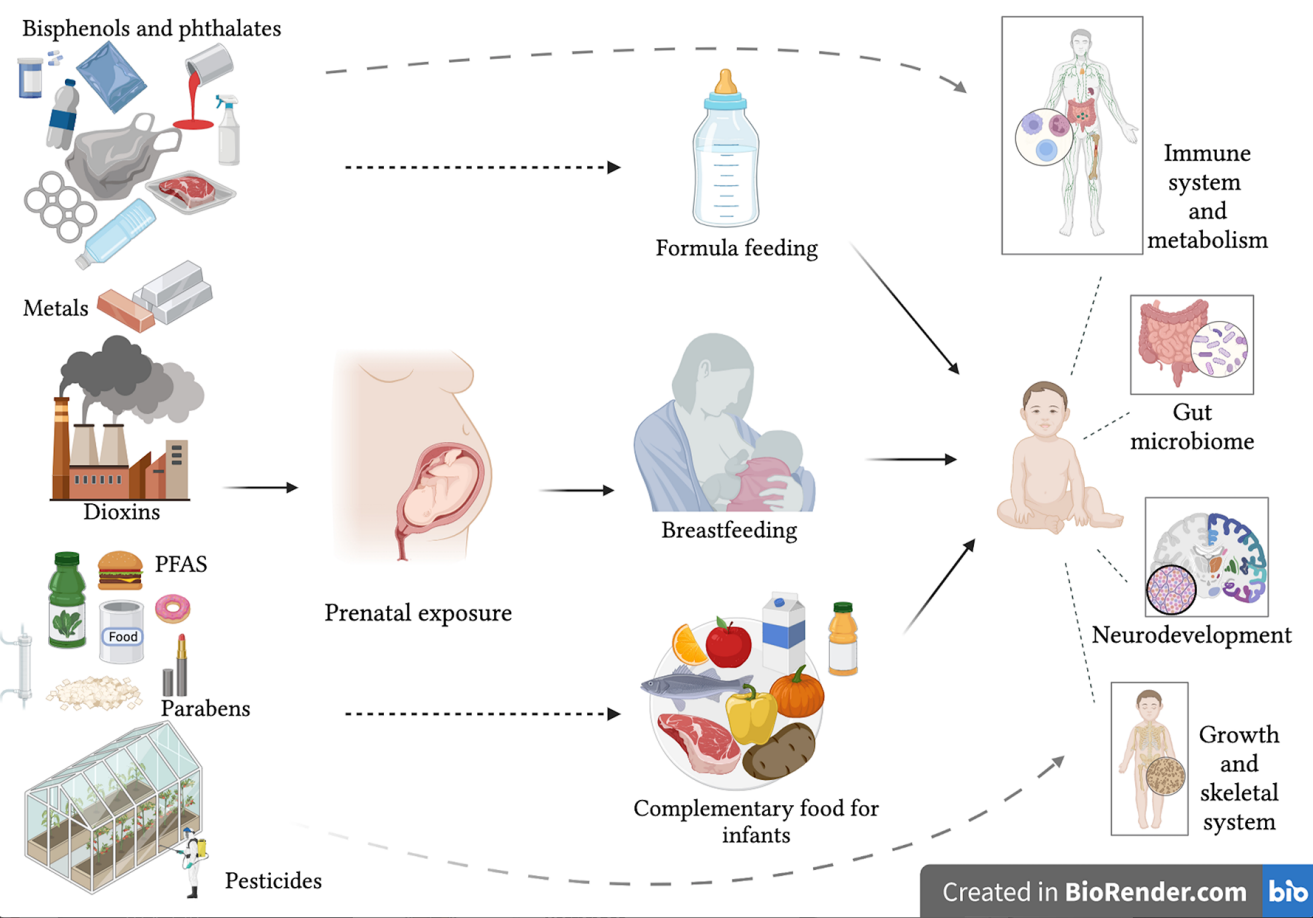
Possible consequences on infant health of exposure to endocrine disrupting chemicals with early life nutrition.

In early life any contamination of breast milk, and complementary feeding can play a role on immune response and GM development with effects on metabolism, on development of inflammatory diseases and on future health (Figure 1). The association between unbalanced microbiota diversity or dysbiosis and possible biological mechanisms responsible for the onset of diseases in different environmental exposure contexts remains largely unknown (19). Ongoing research on the effects of both EDCs and indirect food additives on GM may provide important insights, and correcting changes in the GM could represent an alternative for the treatment and prevention of metabolic diseases and inflammatory responses. Effects of exposure to EDCs can occur in childhood and/or adulthood, and some may be transient. Overall, there is, however, an increased need for more awareness, and further studies are warranted to improve our understanding of pathogenic mechanisms. Furthermore, prevention campaigns should be designed to limit exposure before beginning pregnancy. The ongoing European LIFE-MILCH project,^1^ focuses on detecting EDCs in breast milk and their effects on infants’ growth, adiposity and development from birth up to 12 months of age, and at establishing a clear risk assessment model to prepare and disseminate safety guidelines to reduce and prevent exposure to these chemical substances. Ultimately the aim is to build a targeted and useful prevention campaign to protect and improve breastfeeding (154).

## Author contributions

MS and CC conceived, contributed in the writing and revision of the entire manuscript. A-MS and RR, reviewed the literature and participated in writing the draft. GG revised and wrote the manuscript. A-MS prepared the figure. MS and A-MS prepared the table. All authors contributed to the article and approved the submitted version.

## Conflict of interest

The authors declare that the research was conducted in the absence of any commercial or financial relationships that could be construed as a potential conflict of interest.

## Publisher’s note

All claims expressed in this article are solely those of the authors and do not necessarily represent those of their affiliated organizations, or those of the publisher, the editors and the reviewers. Any product that may be evaluated in this article, or claim that may be made by its manufacturer, is not guaranteed or endorsed by the publisher.

## Footnotes

^1^ www.lifemilch.eu
